# Symbiotic Association with *Mycoplasma hominis* Can Influence Growth Rate, ATP Production, Cytolysis and Inflammatory Response of *Trichomonas vaginalis*

**DOI:** 10.3389/fmicb.2016.00953

**Published:** 2016-06-20

**Authors:** Valentina Margarita, Paola Rappelli, Daniele Dessì, Gianfranco Pintus, Robert P. Hirt, Pier L. Fiori

**Affiliations:** ^1^Department of Biomedical Sciences, University of SassariSassari, Italy; ^2^Faculty of Medical Sciences, Institute for Cell and Molecular Biosciences, Newcastle UniversityNewcastle upon Tyne, UK

**Keywords:** *Trichomonas vaginalis*, *Mycoplasma hominis*, ATP, nitric oxide, inflammatory response, endosymbiotic relationship

## Abstract

The symbiosis between the parasitic protist *Trichomonas vaginalis* and the opportunistic bacterium *Mycoplasma hominis* is the only one currently described involving two obligate human mucosal symbionts with pathogenic capabilities that can cause independent diseases in the same anatomical site: the lower urogenital tract. Although several aspects of this intriguing microbial partnership have been investigated, many questions on the influence of this symbiosis on the parasite pathobiology still remain unanswered. Here, we examined with *in vitro* cultures how *M. hominis* could influence the pathobiology of *T. vaginalis* by investigating the influence of *M. hominis* on parasite replication rate, haemolytic activity and ATP production. By comparing isogenic mycoplasma-free *T. vaginalis* and parasites stably associated with *M. hominis* we could demonstrate that the latter show a higher replication rate, increased haemolytic activity and are able to produce larger amounts of ATP. In addition, we demonstrated in a *T. vaginalis*-macrophage co-culture system that *M. hominis* could modulate an aspect of the innate immuno-response to *T. vaginalis* infections by influencing the production of nitric oxide (NO) by human macrophages, with the parasite-bacteria symbiosis outcompeting the human cells for the key substrate arginine. These results support a model in which the symbiosis between *T. vaginalis* and *M. hominis* influences host-microbes interactions to the benefit of both microbial partners during infections and to the detriment of their host.

## Introduction

*Trichomonas vaginalis*, an obligated extracellular mucosal microbial parasite, is the causative agent of trichomoniasis, the most common cellular, curable, sexually transmitted disease worldwide (Hobbs et al., 2008). This infection affects millions of women and men every year and the World Health Organization estimated that in 2008 there were 276.4 million new cases (WHO, 2012). In women, the infection ranges from asymptomatic to a severe vaginitis, and can be associated with important complications such as pelvic inflammatory disease, sterility, pregnancy and postpartum problems (Petrin et al., 1998; Hobbs et al., 2008; Fichorova, 2009; WHO, 2012; Hirt and Sherrard, 2015; Kissinger, 2015). In men, infections occur mainly without overt symptoms, complicating diagnosis and control of the parasite (Petrin et al., 1998; Hobbs et al., 2008; Fichorova, 2009; Hirt and Sherrard, 2015; Kissinger, 2015). Moreover, trichomoniasis has been associated with invasive cervical cancer (Yap et al., 1995; Viikki et al., 2000) and more recently with aggressive prostate cancers (Sutcliffe et al., 2006; Twu et al., 2014). Arguably one of the most dramatic impacts of *T. vaginalis* infections on human health is through its strong epidemiological association with HIV transmission and acquisition (Kissinger, 2015).

Trichomoniasis is linked with dysbiosis of the urogenital tract microbiota and unbalanced inflammatory responses. Among symptomatic patients important tissue damages can be observed (Hirt and Sherrard, 2015; Kissinger, 2015). It is through these different impacts on the mucosal surfaces that *T. vaginalis* is thought to facilitate HIV entry and transmission (Fichorova et al., 2013; Hirt and Sherrard, 2015; Kissinger, 2015). Indeed, there are an increasing number of studies supporting a strong epidemiological association between *T. vaginalis* and HIV (Guenthner et al., 2005; McClelland et al., 2007; Thurman and Doncel, 2011; Kissinger, 2015). Epidemiologic association between *T. vaginalis* and other human infecting viruses are also likely, including HPV and HSV (Tao et al., 2014; Kissinger, 2015). Other viruses of interest to consider in this context are *T. vaginalis* infecting viruses member of the Totoviridae, which in contrast to human infecting viruses can replicate in the parasite, these are transmitted vertically (*T. vaginalis* viruses, TVV) (Goodman et al., 2011). Significantly the combination *T. vaginalis*-TVV could modulate the pathobiology of the parasite by modifying parasite gene expression and synergistically stimulating pro-inflammatory innate responses (Fichorova et al., 2012, 2013; Hirt and Sherrard, 2015; Kissinger, 2015).

*Trichomonas vaginalis* can internalize members of the bacterial microbiota through phagocytosis, potentially contributing to vaginal dysbiosis possibly by killing differentially members of the protective lactobacilli bacteria and by hosting and transmitting opportunistic, pathobionts, Mollicutes species (Bär et al., 2015). Indeed the presence of *T. vaginalis* is correlated with a low abundance of protective lactobacilli and higher proportions of *Mycoplasma, Prevotella* and other bacterial species typically associated with bacterial vaginosis (BV) (Brotman et al., 2012; Martin et al., 2013; Bär et al., 2015; Hirt and Sherrard, 2015). Notably, *T. vaginalis* has shown a strong clinical association with two different species of Mollicutes: *Mycoplasma hominis* (Koch et al., 1997), and more recently with *Candidatus Mycoplasma girerdii* (Martin et al., 2013; Fettweis et al., 2014). Although little is currently known about the medical importance of *Candidatus M. girerdii* (Fettweis et al., 2014), *M. hominis* is considered a commensal organism of the lower urogenital tract that is increasingly recognized as an opportunistic pathogen, causing genital infections linked with several pregnancy and postpartum complications including spontaneous abortion, endometritis and low birth weight (Capoccia and Baud, 2013). Intriguingly a number of studies have demonstrated a symbiotic interaction between *T. vaginalis* and *M. hominis*. In fact more than 80% of *T.vaginalis* isolates tested so far are naturally infected by *M. hominis*, independently from their geographic origin (Rappelli et al., 1998, 2001; Dessì et al., 2005, 2006). The interaction between *T. vaginalis* and *M. hominis* is the first symbiosis described involving two obligate human mucosal microbes that can produce independent diseases in the same anatomical area. However, the exact nature and fundamental aspects of this microbial symbiosis, and in particular its potential impact on the human host, still have to be clarified.

**Figure 1:**
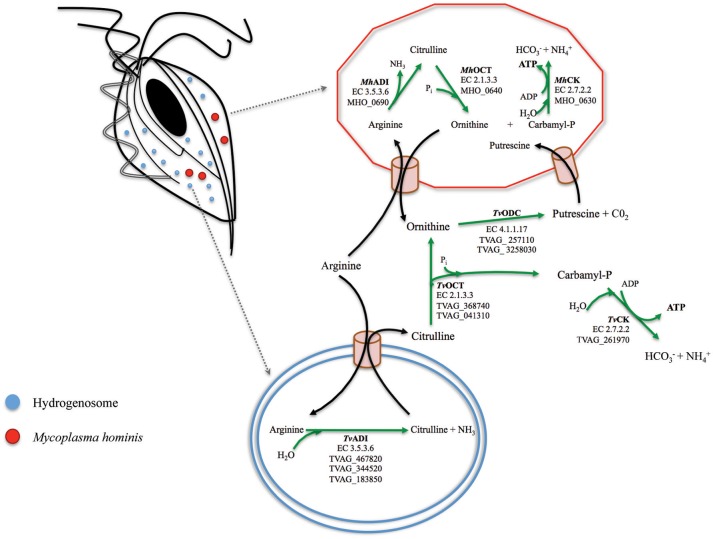
**A model for the arginine dihydrolase pathway in the ***T. vaginalis*** and ***M. hominis*** symbiosis**. The enzymes involved in the ADH pathway in *T. vaginalis* (flagellated cell on the top left with one of its hydrogenosome highlighted at the bottom where ADI is located; Morada et al., 2010) and in *M. hominis* (red cell on the top right) have been reported for both organisms (Shah and Swiatlo, 2008; Morada et al., 2010). The locus tags TVAG_467820; TVAG_344520; TVAG_183850 (*T. vaginalis*) and MH_0690 (*M. hominis*) for the genes encoding the enzymes of the ADH pathway are indicated. Arginine is hydrolytically cleaved to citrulline by arginine deaminase (ADI, EC 3.5.3.6), and citrulline undergoes phosphorolysis by catabolic ornithine carbamiltransferase (OCT, EC 2.1.3.3) to ornithine and carbamyl phosphate. Ornithine is converted in putrescine by ornithine decarboxylase (ODC, EC 4.1.1.17) and carbamyl phosphate is broken down by a catabolic carbamate kinase enzyme (CK, EC 2.7.2) to bicarbonate and ammonia with concomitant production of ATP.

In recent years some aspects of the association between the two pathogens have been characterized. *M. hominis* has the capability to associate with *T. vaginalis* both at the cell surface and intracellularly, and to replicate in a coordinated fashion (Dessì et al., 2005). Moreover, mycoplasmal infection can be passed from naturally mycoplasma-infected trichomonads to naturally mycoplasma-free trichomonad isolates and to human cervical cells (Rappelli et al., 2001). These data suggest that *T. vaginalis* could play the role of a “Trojan horse” for the bacterium during parasite infections, since the intracellular location of *M. hominis* in *T. vaginalis* cells may contribute to protect the bacteria from the host immune system and possibly from antibiotics unable to cross cellular membrane (Dessì et al., 2005, 2006). In addition, *M.hominis* is resistant to anti-trichomonad drugs as metronidazole and tinidazole. Data from *in vitro* experiments indicate that *M. hominis* induces higher level of *T. vaginalis* cytopathogenicity to vaginal epithelial cells, amoeboid transformation rates and yeast phagocytosis (Vancini et al., 2008) and that the presence of the bacteria synergistically up-regulates the pro-inflammatory response of human macrophages exposed to *T. vaginalis* (Fiori et al., 2013). *Trichomonas vaginalis* and *M. hominis* also show a common biochemical pathway, the Arginine Dihydrolase (ADH) pathway (Yarlett et al., 1996). In both organisms, arginine is converted to ornithine and ammonia through the enzymes arginine deiminase (ADI), catabolic ornithine carbamyltransferase (OCT) and carbamate kinase (CK), and finally ATP is generated by depletion of nitrogen from amino acids. ADI are important enzymes implicated in several bacterial infections (Ryan et al., 2009; Fulde et al., 2011) and recently have been shown to be involved in virulence and pathogenicity of the mucosal gut microbial parasite *Giardia duodenalis* (Banik et al., 2013). In *T. vaginalis* ADI is localized in hydrogenosomes while the other enzymes of the ADH pathway are cytosolic (Figure 1; Morada et al., 2011). This is the major energetic pathway of *M. hominis* (Pereyre et al., 2009), while *T. vaginalis* uses it to acquire up to the 10% of its energy requirements (Yarlett et al., 1996). Notably, *T. vaginalis* co-cultured with *M. hominis* showed an increase in arginine consumption and in ornithine and putrescine production, as compared to mycoplasma-free *T. vaginalis* (Morada et al., 2010). The benefit for *M. hominis* could be an increased supply of putrescine, which the bacteria cannot synthesize and that is important for normal cellular growth (Shah and Swiatlo, 2008). For *T. vaginalis* the physiological advantage of this metabolic interaction is debatable but, given that *T. vaginalis* in symbiosis with *M. hominis* consume larger amounts of free arginine compared to trichomonads alone (Morada et al., 2010), a metabolic association could contribute to down-regulate arginine dependent host innate defenses. Indeed, depletion of arginine from the extracellular environment is considered a virulence mechanism allowing several pathogens to interfere with antimicrobial nitric oxide (NO) production by human macrophages (Bronte and Zanovello, 2005; Das et al., 2010; Gogoi et al., 2015).

The symbiosis between these two human associated cellular microbes, both with pathogenic potential, represents a fascinating example of poly-microbial infection, highlighting the increasingly recognized complexity of host-microbes interactions with important implications for how basic research on host-parasite interactions is performed and that ultimately will impact on future diagnostics and therapeutic strategies for sexually transmitted infections (Clemente et al., 2012; Fichorova et al., 2012, 2013; Bär et al., 2015; Hirt and Sherrard, 2015; Kissinger, 2015). Here we aimed at gaining further insights into the potential influence of *T. vaginalis-M. hominis* symbiosis on the physiology and pathobiology of the parasite. We studied *in vitro* the effects of *M. hominis* on *T. vaginalis* growth rate and ATP concentration and investigated how the presence of *M. hominis* may influence the hemolytic activity of the parasite, since recently was demonstrated that *T. vaginalis* strain G3 has the higher ability to induce red blood cells (RBCs) lysis compared with other 25 trichomonas strains (Lustig et al., 2013). Finally, we studied how the association between *T. vaginalis* and *M. hominis* may influence NO production in a human monocytic cell line.

## Materials and methods

### Materials and reagents

Cell culture media, RPMI 1640 with (1.15 mM L-Arginine) or without L-Arginine were obtained from GIBCO and Sigma-Aldrich, respectively. Lipopolysaccharides from *Escherichia coli* 055:B5, recombinant human IFN-γ produced in *E.coli*, the Griess reagent and Phorbol-12-myristate -13- acetate (PMA) were all purchased from Sigma-Aldrich. Fetal bovine serum (FBS) was purchased from GIBCO.

### Parasites and culture conditions

*T. vaginalis* reference strain G3, naturally mycoplasma-free, and isogenic G3 strain experimentally associated with *M. hominis* isolate (MPM2) were cultured by daily passages at 1:16 in Diamond's TYM (trypticase, yeast extract and maltose) medium supplemented with 10% FBS at 37°C in a 5% CO_2_ atmosphere (Diamond, 1957). *M. hominis* cells (named MPM2) were isolated from the naturally infected *T. vaginalis* strain TvMPM2 as previously described (Rappelli et al., 2001), maintained in BEA medium (2.2% heart infusion broth;, 15% horse serum; 1.9% yeast extract; 40 IU/ml benzylpenicillin; 0.23% L-arginine; 0.0023% phenol red, pH 7.2) (Andersen et al., 1987).

In order to obtain an isogenic mycoplasma-infected trichomonad strain, *T. vaginalis* G3 was stably infected with *M. hominis* MPM2 as previously described (Morada et al., 2010). Briefly, 1 ml of an overnight culture of *M. hominis*, corresponding to approximately 10^9^ color-changing units (CCU), was added daily to a 10-ml mid-log phase culture of the *M. hominis*-free *T. vaginalis* isolate G3 for 5 days in TYM complete medium. Parasites were subsequently cultivated for 10 days with 1:16 daily passages in TYM complete medium. Stable infection by *M. hominis* was then assessed by PCR with specific primers (Blanchard et al., 1993) and by isolating in liquid and solid BEA medium *M. hominis* from *T. vaginalis* cultures. The stably infected *T.vaginalis* isolate has been named G3-MPM2.

Growth curves of *M. hominis*-free and isogenic *M. hominis*-infected protozoa were compared. Briefly, 400,000 parasite cells were inoculated in 10 ml of TYM medium and incubated at 37°C. Cell counts were recorded at 2, 6, 9, 12, 18, 24, 32 h after inoculation. The experiments were performed three times, in triplicate.

In order to avoid cross contamination, mycoplasma-free and isogenic *M. hominis*-infected *T. vaginalis* G3 were separately grown in distinct incubators, in all experiment described.

### Haemolytic activity of *M. hominis*-infected strains

*T. vaginalis* G3 associated with *M. hominis* isolate (MPM2) was contrasted with isogenic *T. vaginalis* G3 naturally mycoplasma-free, in order to evaluate haemolytic activity. Haemolysis assays were performed as previously described (Fiori et al., 1993). Briefly, RBCs were collected from healthy human donors; erythrocytes were then washed three times in PBS and immediately used. Parasites in exponential growth phase were washed twice in PBS and resuspended to a density of 2 × 10^6^ in PBS + 15 mM maltose (PBS-M). *T. vaginalis* G3 and G3 associated with MPM2 were incubated at 37°C with washed erythrocytes in a ratio 1:30 in PBS-M. The hemoglobin released after incubation with red RBCs for different times ranging from 90 to 210 min, was evaluated by spectrophotometric analysis at 546 nm adsorbance. Haemolytic capacity of *M. hominis*-infected G3 strains was compared to parental uninfected isogenic *T. vaginalis* G3.

In order to detect if *M. hominis* itself could be able to lyse RBCs, 10 ml of overnight culture of *M. hominis*, corresponding to approximately 10^10^ color-changing units (CCU) were centrifuged, washed three times in PBS, resuspended in 1 ml PBS-M and incubated with 2 × 10^6^ RBCs at the same time intervals.

### Production of ATP in *M. hominis*-infected and *M. hominis*-free *T. vaginalis*

The amount of intracellular ATP produced by mycoplasma-free and mycoplasma-infected *T. vaginalis* G3 was evaluated in three different phases of growth: lag, exponential, and stationary phase. Furthermore, the amount of ATP produced by microorganisms grown in media additioned with 0.1 mM or 0.5 mM L-Arginine was also compared. A total of 15,000 cells of the parasites in exponential growth phase for each tested condition was harvested and centrifuged at 4000 × g for 10 min and the amount of ATP analyzed in 50 μl by using CellTiter-Glo Luminescent Cell Assay (Promega) according to the manufacturer's instructions.

### Cell cultures and THP-1 differentiation

Human monocytic cell line THP-1 (ATCC® TIB202™) was cultured in RPMI 1640 or RPMI 1640 without L-Arginine, supplemented with 10% FBS at 37°C in a humidified atmosphere containing 5% CO_2_ and maintained at 5 × 10^5^ cells/ml. THP-1 cells were seeded at 5 × 10^5^ cells per well in 24-well plates and differentiated to a macrophage-like phenotype (dTHP-1) by stimulation with 50 ng/ml PMA for 18 h. Medium was replaced and cells were maintained with two daily medium changes for further 5 days before co-culture with protozoa.

### Production of NO by human macrophages stimulated with *M. hominis*-infected and *M. hominis*-free *T. vaginalis*

dTHP-1 cells seeded at 5 × 10^5^ cells per well in 24-well plates, were stimulated with *T. vaginalis* G3 or G3-MPM2 with 1:10 *T. vaginalis*/macrophage ratio, in presence of 100 Units/ml INF-γ (Park et al., 1997) in RPMI 1640 (standard 1.15 mM arginine). Untreated dTHP-1 and dTHP-1 stimulated cells with 1 μg/ml of LPS were used as negative and positive controls respectively.

In a second group of experiments, dTHP-1 were depleted of their intracellular L-arginine store by cultivation in RPMI 1640 without L-Arginine for 60 h; dTHP-1 cells were then co-incubated with *T. vaginalis* G3 and with G3-MPM2 in RPMI 1640 containing 0.5 mM and 0.1 mM L-arginine (final concentration).

To evaluate the ability of *M. hominis* alone to induce NO release from macrophages, we have stimulated dTHP-1 with cell-free supernatant of *M. hominis*-infected protozoan cultures, filtered with 0.45 and 0.22 μm pore size filters, containing and not containing bacteria respectively.

The amount of NO produced by dTHP1 cells after stimulation with pathogens was determined measuring the nitrite levels in the culture medium through the Griess reaction (Park et al., 1997). Briefly, culture medium was removed, centrifuged at 4000 × g for 10 min and nitrite levels measured in supernatants. Equal volume of supernatants and Griess reagent (0.1% naphthylenedamine dihydrochloride, 1% sulphanilamide and 2.5% H_3_PO_4_) was incubated for 15 min at room temperature in the dark. The absorbance was measured at 540 nm in an ELISA reader. Nitrite concentration (μM range) was determined using NaNO_2_ as standard.

### Statistical analysis

All experiments were carried out at least in triplicate. Statistical analyses were performed using unpaired Student *t*-test (SigmaPlot version 12.0, from Systat Software, Inc., San Jose California USA and Microsoft Excel; Microsoft, Redmond, Washington, USA). A *p* < 0.05 was considered significant.

## Results

### Effect of *M. hominis* on *T. vaginalis* cultures

**Figure 2:**
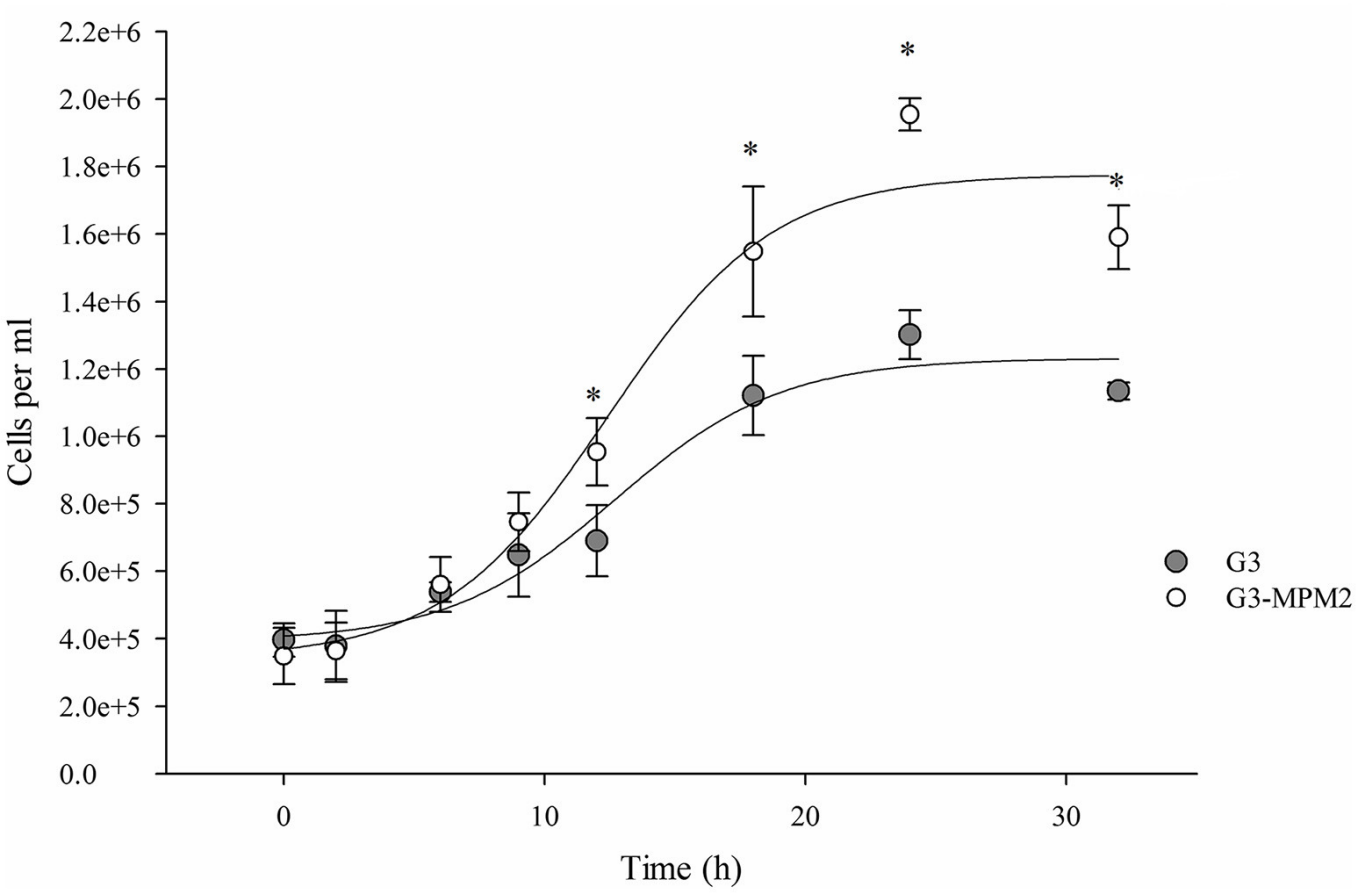
**The influence of ***M. hominis*** MPM2 on ***T. vaginalis*** growth curves**. The starting point for both growth experiments was 400,000 parasite cells/ml and data were collected at indicated times. The rate of replication of *T. vaginalis* associated with *M. hominis* is higher than the parasite alone, suggesting an important influence by the bacteria on the parasite physiology. The results represent the average of three independent experiments and error bars represent standard deviations. Statistical significance was tested by Student *t*-test and ^*^p < 0.05 was considered significant. The growth curves for *T. vaginalis* G3 and *T. vaginalis* in symbiosis with *M. hominis* MPM2 were obtained by fitting the data using a non-linear regression.

The growth rate of *M. hominis*-free (G3) and *M. hominis*-infected *T. vaginalis* (G3-MPM2) in complete Diamond's TYM medium, routinely used to grow the parasite, was compared. Cells were cultured for a total of 32 h and *T. vaginalis* infected with *M. hominis* were characterized by ~ 20% faster growth rate than mycoplasma-free parasites, leading to a ~40% higher cell densities in the stationary phase (Figure 2).

### Effect of *M. hominis* on haemolytic activity of *T. vaginalis*

**Figure 3:**
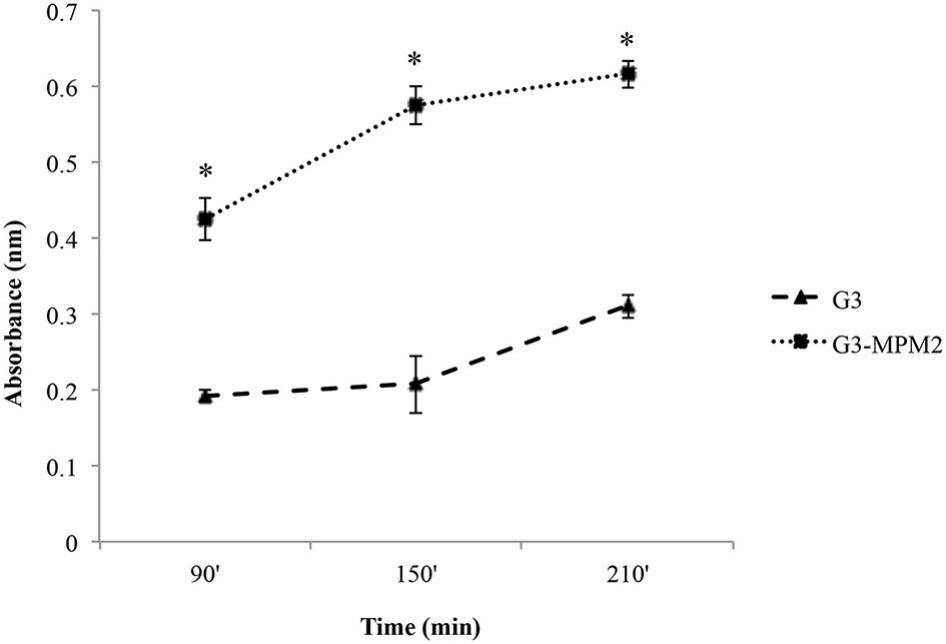
**The impact of ***M. hominis*** symbiosis on ***T. vaginalis*** haemolytic activity**. Haemolytic activity exerted by *M. hominis*-infected *T. vaginalis* G3 and uninfected *T. vaginalis* G3 was compared by analysing hemoglobin released by RBCs through spectrophotometric analysis (reading at 546 nm absorbance). The values define hemoglobin released by RBCs upon contact with pathogens and represent average and error bars standard deviations for three independent experiments. Statistical significance was tested by Student *t*-test and ^*^ indicate significant (*p* < 0.05) variations compared to parasites without *M. hominis*.

We tested whether *M. hominis* might influence the hemolytic properties of *T. vaginalis in vitro*, by using a haemolysis assay (Fiori et al., 1993). The amount of hemoglobin released by RBCs upon contact with pathogens was evaluated at time intervals ranging from 90 to 210 min. The protozoa infected with *M. hominis* MPM2 were characterized by higher haemolytic activities compared to the mycoplasma-free isogenic *T. vaginalis* G3 strain (Figure 3).

Control experiments to exclude direct haemolytic activity of *M. hominis* MPM2, clearly indicated that the bacteria were unable to lyse RBCs.

### *T. vaginalis* symbiotically-associated with *M. hominis* produces higher amounts of ATP

**Figure 4:**
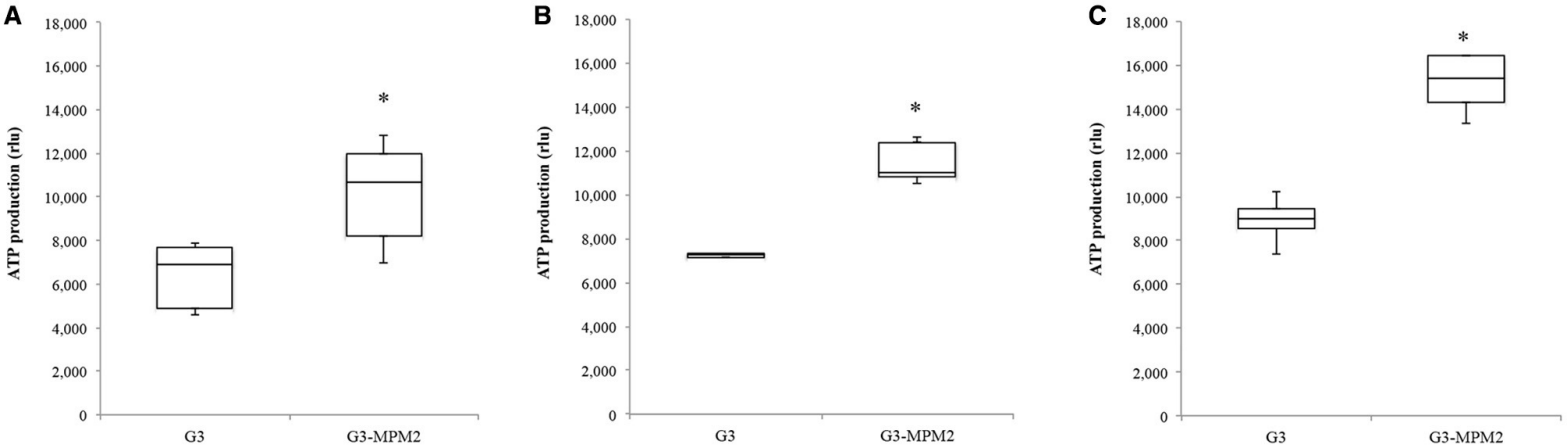
**Intracellular amount of ATP produced by ***T. vaginalis*** and ***T. vaginalis*** infected with ***M. hominis*** in different growth phases**. (**A**: lag phase; **B**: exponential phase; **C**: stationary phase). The amount of ATP produced by the *T. vaginalis*-*M. hominis* (G3-MPM2) consortium is higher than that produced by *T. vaginalis* (G3) alone in all growth phases. The total amount of ATP, corresponding to the number of cells tested (15,000 cells), is proportional to luminescent signal generated by luciferase reaction. RLU corresponds to Relative Light Units. Bars represent the mean ±S.D. of at least three independent experiments. Data were analyzed by Student's *t*-test. ^*^p < 0.05 when intracellular amount of ATP produced by G3-MPM2 is compared to amount of ATP produced by G3.

**Figure 5:**
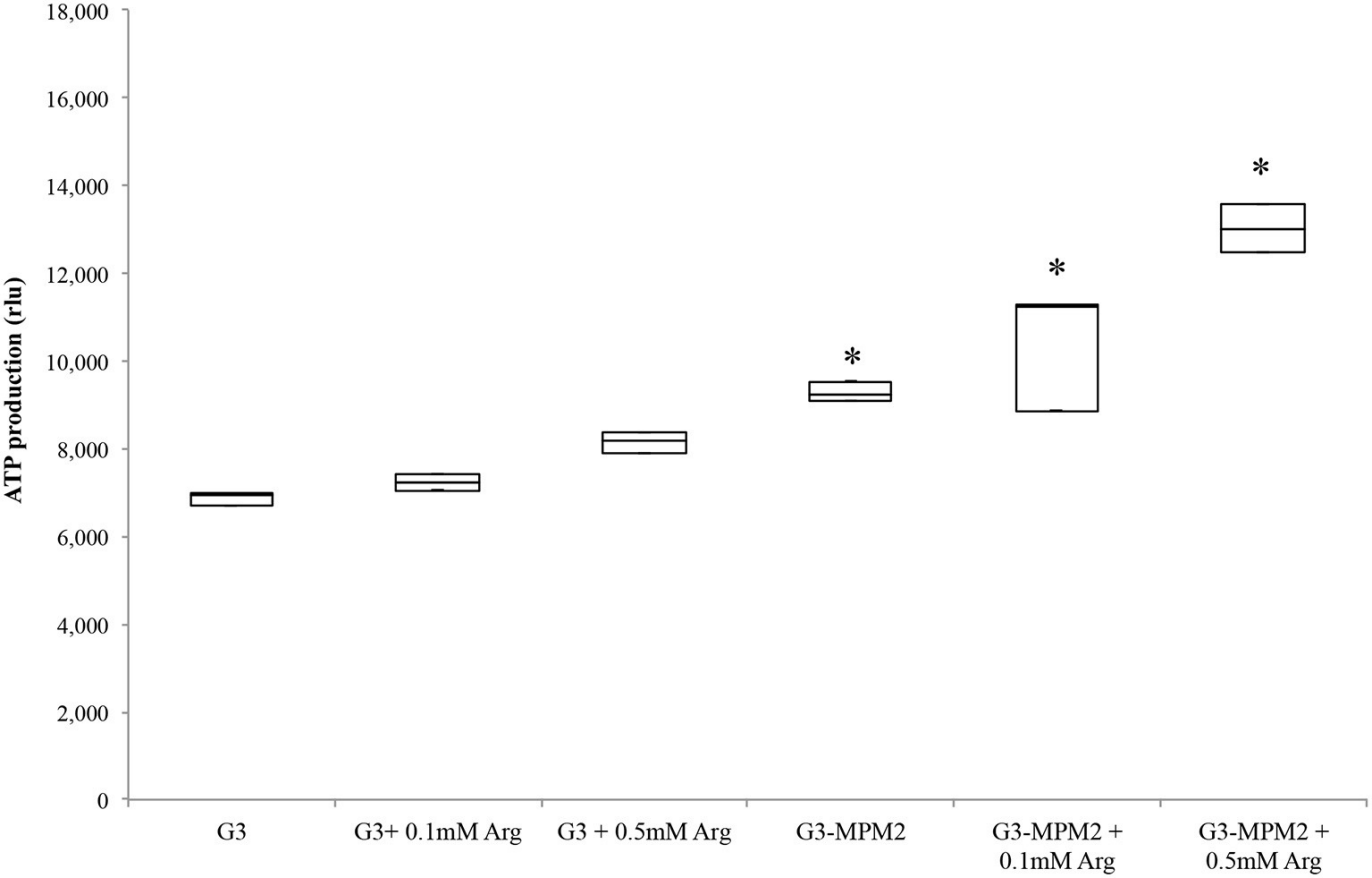
**ATP produced by ***T. vaginalis*** and ***T. vaginalis–M. hominis*** growth in media with different concentration of arginine**. The microorganisms were grown in media supplemented with different concentrations of arginine, compared with the same strains cultured in normal Diamond's medium. *M. hominis* infected trichomonads cells lead to an approximately 2-fold increase in ATP production compared to *T. vaginalis* grown alone, both in normal Diamond's medium and in medium complemented with higher concentration of arginine. G3, naturally mycoplasma-free *T. vaginalis*; G3/MPM2, symbiotically-associated *T. vaginalis* G3 and *M. hominis* MPM2. Analyzes represent averages of three independent triplicate experiments. Data were analyzed by in R (^*^p < 0.05) using a Linear mixed-effects model fit by REML.

Since *T. vaginalis* can use arginine as an additional source of energy via the ADH pathway (Morada et al., 2010), and *M. hominis* utilizes the same metabolic pathway as its main source of ATP (Pereyre et al., 2009), we have investigated the possibility that *M. hominis* influences the amount of ATP produced in the context of the *T. vaginalis-M. hominis* consortium. The amount of ATP produced by *T. vaginalis* G3-MPM2 is approximately 2-fold higher than that produced by isogenic G3 in all growth phases for the tested growth conditions (Figure 4). We asked next whether arginine concentration in growth medium could influence ATP production in both *M. hominis* infected and uninfected *T. vaginalis*. The experiments were performed adding 0.1 mM or 0.5 mM L-Arginine to the growth medium. The ATP produced by *T. vaginalis* G3 cells grown in medium supplemented with arginine is only marginally increased compared to parasites grown in the standard medium. In contrast, additional arginine leads mycoplasma-infected parasites to produce significantly higher amount of ATP (Figure 5).

### *M. hominis*-infected *T. vaginalis* competes with host macrophages for arginine uptake and down regulates NO production

**Figure 6:**
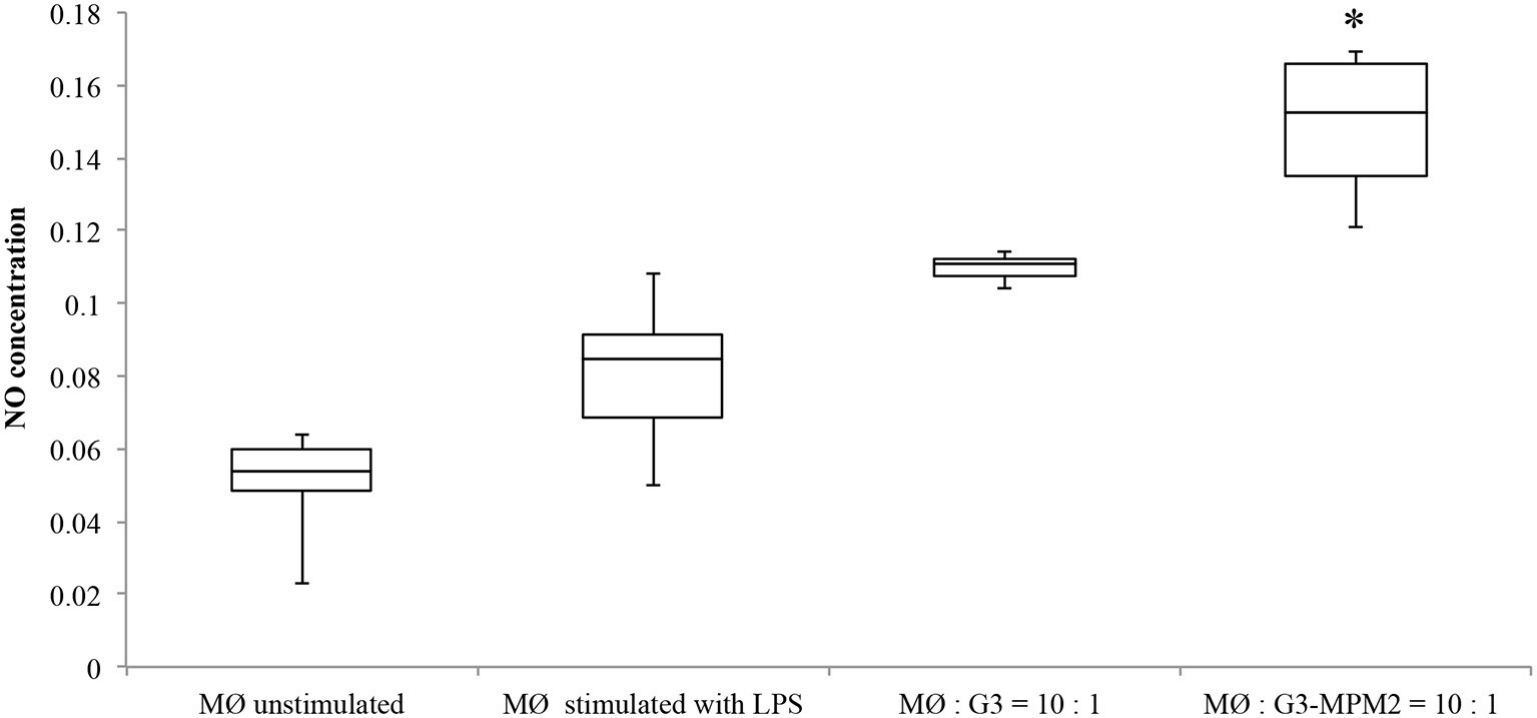
**NO produced by human macrophages after co-culture with pathogens**. The amount of NO produced by dTHP-1 cultivated RPMI 1640 containing standard arginine concentration (1.15 mM) after 24 h of exposure to *T. vaginalis* G3 and *T. vaginalis* G3-MPM2 was compared. Experiments were carried out with 10:1 macrophages to parasites ratio. dTHP-1 cells produce higher amount of NO in response to stimulation with *T. vaginalis* G3-MPM2 (MØ:G3/MPM2) compared to *M. hominis*-free isogenic *T. vaginalis* (MØ:G3). The values represent averages of at least three independent experiments. Data were analyzed by Student's *t*-test (^*^p < 0.05). dTHP-1 cells unstimulated and stimulated with LPS and INFγ represent negative and positive controls respectively.

Depletion of host arginine is considered as a microbial virulence strategy preventing toxic NO formation by macrophages. Hence we evaluated NO production in human macrophages stimulated with *T. vaginalis*-associated and non-associated with *M. hominis*. THP-1 cells were differentiated to a macrophage-like phenotype by PMA (dTHP-1) and were thereafter incubated with *T. vaginalis* G3 or *T. vaginalis* G3-MPM2, with 1:10 *T. vaginalis*/macrophage ratio for both conditions in medium with high concentration of arginine (1.15 mM in RPMI 1640). The supernatants were harvested 24 h after microbial stimulation and the amount of NO was evaluated. We observed higher secretion of NO upon dTHP-1 stimulation with *T. vaginalis* G3-MPM2, compared to mycoplasma-free isogenic trichomonads (Figure 6).

To evaluate the effects of *M. hominis* alone, cell-free supernatants from MPM2-infected *T. vaginalis* filtered with 0.45 μm, and 0.22 μm pore size filters (that completely eliminates bacteria) were incubated with THP-1 cells, as previously described. After 24 h stimulation, macrophages incubated with supernatants filtered with 0.45 μm pore size filters, produced NO amounts comparable with stimulation with LPS and INF-γ range from 0.07 to 0.08 μM), while THP-1 cells incubated with 0.22 μm pore size filtered supernatants, were not stimulated (data not shown).

These data indicate that bacterial symbionts up-regulate NO production by macrophages, reminiscent of the synergistic effect of the *T. vaginalis*-*M. hominis* symbiosis on macrophages pro-inflammatory response *in vitro* (Fiori et al., 2013).

**Figure 7:**
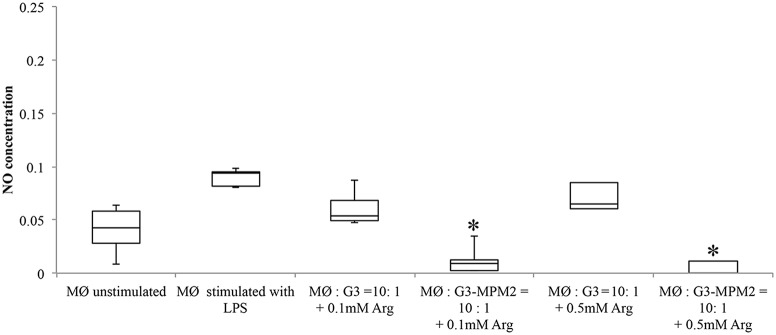
**Effect of arginine on NO production by macrophages stimulated with pathogens**. Arginine-depleted dTHP-1 (following cultivation in arginine-free RPMI 1640), were incubated for 24 h with MPM2-free (MØ:G3), and MPM2-infected *T. vaginalis* (MØ:G3/MPM2) in presence of two different arginine concentration (0.1 and 0.5 mM respectively). Experiments were carried out with 10:1 macrophages to parasites ratio. There is a significant reduction of NO produced by dTHP-1 incubated with *T. vaginalis* G3-MPM2. Results demonstrate that the metabolism of *M. hominis* contributes to consume the arginine in the medium. Analyzes represent averages of three independent triplicate experiments. Data were analyzed in R (^*^p < 0.05) using a Linear mixed-effects model fit by REML.

In order to test if arginine metabolism of symbionts *M. hominis* could contribute to subtraction of this amino acid and, by consequence, inhibit NO production by macrophages, THP-1 cells were differentiated (dTHP-1 cells) and cultured in arginine-free media to deplete arginine intracellular stores. dTHP-1 were then stimulated with *T. vaginalis* or *T. vaginalis*-*M. hominis* consortium in presence of either 0.1 or 0.5 mM of arginine. These two concentrations were chosen as the vaginal concentration of arginine has been reported to be ~0.2 mM and reduced to undetected level during *T. vaginalis* infections or BV (Chen et al., 1982). In our experimental conditions we observed a strong decrease in production of NO by macrophages stimulated with *T. vaginalis* G3-MPM2, especially in presence of the lower arginine concentrations (Figure 7).

The amount of NO released by macrophages after stimulation with filtered supernatants from mycoplasma-free and mycoplasma-infected *T. vaginalis* in arginine-free media is undistinguishable compared with NO stimulation in medium with arginine (data not shown).

These results suggest that *T. vaginalis* associated with *M. hominis* could compete with macrophages to uptake free arginine from the environment, leading to a significant down-regulation of NO production.

## Discussion

It is increasingly recognized that complex poly-microbial interactions involving the parasite *T. vaginalis*, its viruses (TVV) (Goodman et al., 2011), members of the vaginal microbiota and bacteria associated with BV, including a range of bacteria with pathogenic potentials such a *M. hominis*, can influence the mucosal inflammatory tone and general integrity (Rappelli et al., 2001; Brotman et al., 2012; Fichorova et al., 2013; Bär et al., 2015). This is thought to significantly increase both transmission rates of important human viral STI including HIV and HPV (Guenthner et al., 2005; McClelland et al., 2007; Thurman and Doncel, 2011; Fichorova et al., 2013; Tao et al., 2014; Hirt and Sherrard, 2015; Kissinger, 2015) and contribute to adverse pregnancy outcomes (Fichorova, 2009; Hirt and Sherrard, 2015). In this scenario the capacity of *T. vaginalis* and *M. hominis* to enter in a unique form of symbiosis, combined with data supporting a model of co-pathogenesis (Vancini et al., 2008; Fiori et al., 2013) and metabolic symbiosis (Morada et al., 2011) is of particular interest and led us to further investigate how *M. hominis* associated with *T. vaginalis* might influence the parasite physiology and the dynamic of host-parasite-bacteria interactions.

We establishing an experimental parasite-bacteria consortium with a *M. hominis* clinical isolate (MPM2) to performed comparative growth experiments in rich medium. We demonstrated a significant higher growth rate in the log phase and cell density in the stationary phase for the parasite-bacteria consortium compared to the parent isogenic *T. vaginalis* G3 strain grown over the course of 32 h. Notably a period of adaptation was required (4 weeks) for the parasite-bacteria consortium to develop a stable symbiotic association, with the initial growth of the parasite being reduced during the early interactions between parasite and bacteria.

Vancini and colleagues demonstrated that *T. vaginalis* naturally infected by *M. hominis* were characterized by a higher level of cytopathogenicity *in vitro*, compared to protozoa alone (Vancini et al., 2008). To avoid any strain-to-strain variability, we performed haemolysis experiments by using isogenic protozoa with and without *M. hominis* and RBCs as target cells and our data confirm that mycoplasmas enhance the parasite cytopathogenicity, measured through the haemolytic properties of *T. vaginalis in vitro*. These results could be explained by the up-regulation of *T. vaginalis* haemolysis due to the presence of the symbiotic bacteria; for example by increasing the secretion of *T. vaginalis* pore forming proteins (Fiori et al., 1996; Hirt et al., 2011), or by increasing the adhesion of the parasite to RBCs in the presence of the *M. hominis*. The characterization of the molecules and mechanisms mediating haemolysis will be required to shed light on these different hypotheses.

The observed boost in parasite growth rate and the higher cell density in the log phase are consistent with a metabolic symbiosis putatively based on arginine catabolism (Morada et al., 2011) leading to an overall more efficient exploitation of metabolites from the medium by the combined activity of the symbionts. Arginine is rapidly depleted from the medium during *T. vaginalis* cultivation (Linstead and Cranshaw, 1983; Zuo et al., 1995) and it is degraded by ADH pathway, leading to ornithine and putrescine production and final generation of ATP (Yarlett et al., 1996). The production of ATP by ADH pathway has advantages over glucose fermentation since the end product putrescine is non acidic; recently Huang and colleagues have shown that two enzymes involved in this pathway, catabolic ornithine carbamyltransferase (OCT) and carbamate kinase (CK), are up-regulated in the log growth phase of *T. vaginalis* under glucose restriction (GR), suggesting that this energy–producing pathway is important under GR (Huang et al., 2014). In *M. hominis*, ADH is the major energetic pathway (Pereyre et al., 2009) since arginine is the major source of energy (Pereyre et al., 2009) and it is essential for initiation of bacteria growth (Hahn and Kenny, 1974). The parasite-bacteria consortium, characterized by two ADH pathways of distinct evolutionary origins (Morada et al., 2010), exhibits increased arginine consumption, concomitant with increase of ornithine and putrescine production (Morada et al., 2010). We contrasted the amount of intracellular ATP present in *T. vaginalis* in the presence and absence of *M. hominis* in different growth phases and under different environmental arginine concentrations. The results show higher amounts of ATP in the parasite in the presence of the bacteria compared with *M. hominis*-free parasites, in all growth phases. Interestingly, addition of free arginine to culture medium led the *T. vaginalis*-*M. hominis* consortium to be characterized by a further increase in the amount of ATP per cell. It has been demonstrated that an high ATP content in cell cultures is correlated with an increased growth rate of parasites, including *T. vaginalis* (Miyahira and Takeuchi, 1991), supporting our hypothesis that the presence of *M. hominis* may enhance growth rate of *T. vaginalis* by increasing intracellular ATP. The overall observed increase of ATP per cell in the *T. vaginalis-M. hominis* consortium might be due to ATP produced by mycoplasmas through the ADH pathway, since the increment of ATP production is positively associated with arginine concentration, and the expression of all *T. vaginalis* ADI genes is not up-regulated by the bacterial symbionts (Morada et al., 2010). But as the growth rate of the parasite is boosted, a fraction of the increase of ATP could be directly associated with the parasite itself rather than the bacteria only. Characterizing the potential metabolic interactions between the bacteria and the parasite will be required to establish the origin of the increase of ATP and the link between higher ATP and higher growth rate of the parasite in the *T. vaginalis-M. hominis* consortium.

In human macrophages, arginine is the exclusive amino acid substrate for the production of NO by all isoforms of NO synthase (NOS). Regulating arginine availability is a potential defensive mechanism of pathogens, that can lead to the control of NO production (Popovic et al., 2007). Depletion of arginine is a common strategy used by many pathogens to escape immune response and there are several microbial enzymes, such as arginase and arginine deiminase, that compete for arginine with host NOS (Das et al., 2010). Several studies suggested a role of ADI in microbial virulence (Ryan et al., 2009; Fulde et al., 2011) and recently Banik and colleagues have shown that ADI is implicated in the virulence of *Giardia duodenalis* (Banik et al., 2013). Moreover, Noh and colleagues have proposed a role for *M. hominis* ADI in suppression of NO production caused by macrophage-inducible NO synthase (Noh et al., 2002). The macrophage cytotoxicity against *T. vaginalis* is regulated by NO (Park et al., 1997), as well as by proinflammatory cytokines, such as IL-1, IL-6 and TNFα via NF-kB (Han et al., 2009) and the presence of *M. hominis* is implicated in the robust increase of inflammatory mediators by macrophages (Fiori et al., 2013). Hence, we hypothesized a competition between parasites and macrophages in the uptake of free arginine from the environment. Experiments of co-culture between dTHP-1 cells and *T. vaginalis* associated and non-associated with *M. hominis*, in presence of different concentration of arginine, were performed. Results obtained show that in presence of high concentration of environmental arginine the NO production by macrophages is up regulated by the *T. vaginalis-M. hominis* consortium, confirming our previous data on synergistic effect of the parasite-bacteria consortium on the proinflammatory response (Fiori et al., 2013). On the contrary, when macrophages were depleted of their intracellular arginine stores by cultivation in arginine-free media, and then stimulated with mycoplasma-free and mycoplasma-infected *T. vaginalis* in presence of 0.1 or 0.5 mM of arginine, a strong decrease in NO production was reported in macrophages stimulated with the parasite-bacteria consortium. The competition for free arginine uptake between the *T. vaginalis-M. hominis* consortium and phagocytes, strongly reduce nitric oxide production by macrophages.

These data led us to speculate that symbiosis between *T. vaginalis* and *M. hominis* influence host-microbes interactions to the benefit of both microbial partners during infections. *M. hominis* might play a key role in inflammation during trichomoniasis, by interfering with NO production by human macrophages through outcompeting the human cells for the key substrate arginine.

These hypotheses are strongly supported by recent studies that show how the interaction between *T. vaginalis* and vaginal microbiota potentially influence the outcomes of trichomoniasis. The parasite was found to limit different species of *Lactobacillus*, associated with bacterial vaginosis (Fichorova et al., 2013). It has been shown that the presence of *Lactobacillus gasseri* significantly inhibits *T. vaginalis* adhesion to human cells in strain–specific and contact dependent manners (Phukan et al., 2013). Moreover, *Gardnerella vaginalis* and *Atopobium vaginae*, two common mucosal bacteria associated with bacterial vaginosis, can amplify the pro-inflammatory response to protozoan molecules and to TVV (Fichorova et al., 2013).

Here we studied the effects of a single strain of *M. hominis* in symbiosis with the *T. vaginalis* strain G3, that leads to mutualistic inter-microbial interactions with a potential synergistic impact on their pathobiology on their human host. In order to evaluate whether these results represent a typical outcome of *M. hominis* - *T. vaginalis* interactions, further experiments with additional combinations of bacterial and parasite strains will be required.

In conclusion, these findings taken together support a model in which sinergistic association between vaginal mucosal bacteria and *T. vaginalis* might influence trichomoniasis health sequelae, supporting both the growth of associated pathogenic bacteria and increasing the inflammatory response for disease development.

## Author contributions

PF, VM contributed to the design of the work; PF, RH, PR, DD contributed to interpretation of data; VM, GP, DD contributed to analysis of data; VM, PF, RH wrote the work; PR, DD, GP revised critically the manuscript; all authors approved the final version and agree to be accountable for all aspects of the work.

## Funding

This work was supported by Ministero dell'Istruzione, dell'Università e della Ricerca—PRIN 2012 Grant number 2012WJSX8K_004.

### Conflict of interest statement

The authors declare that the research was conducted in the absence of any commercial or financial relationships that could be construed as a potential conflict of interest.
